# The Transcatheter Closure of Patent Ductus Arteriosus in Extremely Low-Birth-Weight Infants: Technique and Results

**DOI:** 10.3390/jcdd10120476

**Published:** 2023-11-27

**Authors:** Alban-Elouen Baruteau, Alain Fraisse, Gianfranco Butera, Carles Bautista-Rodriguez

**Affiliations:** 1 Department of Pediatric Cardiology and Pediatric Cardiac Surgery, CHU Nantes, Nantes Université, FHU PRECICARE, F-44000 Nantes, France; 2 CIC FEA 1413, INSERM, CHU Nantes, Nantes Université, F-44000 Nantes, France; 3 L’institut du thorax, INSERM, CNRS, CHU Nantes, Nantes Université, F-44000 Nantes, France; 4 UMR 1280, PhAN, INRAE, Nantes Université, F-44000 Nantes, France; 5 Pediatric Cardiology Services, Royal Brompton Hospital, Guy’s & St Thomas’ Foundation Trust, London SW3 6NP, UK; a.fraisse@rbht.nhs.uk (A.F.); c.bautista@rbht.nhs.uk (C.B.-R.); 6 National Heart and Lung Institute, Imperial College, London SW7 2BX, UK; 7 Cardiology, Cardiac Surgery and Heart Lung Transplantation, ERN GUARD HEART: Bambino Gesù Hospital and Research Institute, IRCCS, 00165 Rome, Italy; gianfranco.butera@opbg.net

**Keywords:** patent ductus arteriosus, extremely low-birth-weight infants, premature infant, Doppler echocardiography, outcomes

## Abstract

Persistent patent ductus arteriosus is a very common condition in preterm infants. Although there is no management agreed by consensus, despite numerous randomized controlled trials, hemodynamically significant patent ductus arteriosus increases morbidity and mortality in these vulnerable patients. Medical treatment is usually offered as first-line therapy, although it carries a limited success rate and potential severe adverse events. In recent years, transcatheter patent ductus arteriosus closure has fast developed and become widely accepted as a safe and efficient alternative to surgical ductal ligation in extremely low birth weight infants >700 g, using most often the dedicated Amplatzer Piccolo Occluder device. This article aims to provide an appraisal of the patients’ selection process, and a step-by-step description of the procedure as well as a comprehensive review of its outcomes.

## 1. Introduction

Preterm infants are at a higher risk of complications in the setting of patent ductus arteriosus (PDA). A haemodynamically significant PDA increases the risk of chronic respiratory disease, prolonged assisted ventilation, pulmonary hemorrhage, bronchopulmonary dysplasia, intraventricular hemorrhage, necrotizing enterocolitis, renal impairment and death. PDA closure may be considered in any preterm infant with a haemodynamically significant PDA based on a staging system combining both clinical and echocardiographic criteria [1]. However, whether a PDA should be closed remains a controversial issue, as expectant management for PDA in extremely premature infants is noninferior to early ibuprofen treatment with respect to necrotizing enterocolitis, bronchopulmonary dysplasia, or death at 36 weeks postmenstrual age [2]. A stepwise approach is applied, usually starting with medical treatment using cyclooxygenase inhibitors and/or paracetamol, which offer a moderate success rate of only 60–70% whilst exposing patients to potential major adverse events (MAE) such as renal function impairment, necrotizing enterocolitis, bleeding and developmental delay [3]. Surgical PDA ligation has long been the sole non-pharmacological option, but transcatheter closure with no arterial access, using mainly the Amplatzer Piccolo Occluder (APO; Abbott Structural Heart, Plymouth, MN, USA) has now become widely accepted as a safe and efficient alternative to surgical PDA ligation in infants ≥700 g, with an excellent success rate and a low incidence of periprocedural complications in experienced teams [4]. Based on published data, one can consider that multidisciplinary teams that have performed more than 60 catheter-based cases have gained enough experience to achieve a <5% rate of MAE (Table 1). Pathophysiology, echocardiographic assessment, surgical approaches and anesthesia management are covered in the different articles of this Special Issue and our article is primarily focused on both the technical aspects and the results of transcatheter PDA closure in preterm infants. We herein provide an appraisal of the patients’ selection processes, and a step-by-step description of the procedure as well as a comprehensive review of its outcomes.

## 2. Definition–Epidemiology

PDA is defined as failure of the ductus to close within 72 h postnatally. This is the third most common congenital heart defect (CHD), accounting for 10.2% of CHD, with a prevalence of 1.004 per thousand [12]. It is a very common condition in very low-birth-weight (VLBW, i.e., birth weight < 1500 g) and extremely low-birth-weight (ELBW, i.e., birth weight < 1000 g) infants, with an incidence that is inversely related to gestational age. It is reported in around 50% of infants born under 28 weeks of gestation and/or at a birth weight < 1000 g [13], and 85% of them may close spontaneously [14].

## 3. Indication for Closure and Patient Selection

A haemodynamically significant PDA (hsPDA) is a clinical continuum in which the spectrum of disease ranges from mild to severe, depending on the magnitude of the ductal shunt. The effects of an hsPDA on acute physiological change and short-term clinical outcomes are related to a pulmonary to systemic blood flow imbalance, leading to pulmonary over-circulation and systemic hypoperfusion. PDA closure may be considered in any preterm infant with hsPDA based on a staging system combining both clinical (need for respiratory support or mechanical ventilation or need for high-frequency ventilation, feeding intolerance or necrotizing enterocolitis, acute renal failure, hemodynamic instability or metabolic acidosis) and echocardiographic (transductal diameter > 1.5 mm, left heart volume loading, decreased or absent or reversed diastolic flow in the superior mesenteric artery, middle cerebral artery or renal artery, unrestrictive pulsatile transductal flow) criteria [1,15]. Most centers recommend that echocardiographic criteria should be based on serial consecutive exams.

## 4. Available Devices

Since the initial coil occlusion of preterm PDA [16], a few devices have been used successfully to percutaneously close PDAs in ELBW and VLBW infants, including the Amplatzer Vascular Plug II (AVP II, Abbott, Santa Clara, CA, USA) [17,18,19,20], the Amplatzer Vascular Plug IV (AVP IV, Abbott) [21], the Micro Vascular Plug (MVP, Medtronic, Minneapolis, MN, USA) [22], the Micro Plug Set (Micro Plug, KA Medical, Minneapolis, MN, USA) [23,24] and the Amplatzer Duct Occluder II Additional Sizes (ADOIIAS), renamed as Amplatzer Piccolo Occluder (APO, Abbott) after the seminal US clinical trial [10]. However, the lengths of both AVP-II and AVP-IV may be limiting factors in increasing the risk of descending aorta and/or left pulmonary artery (LPA) obstruction. The MVP, initially designed for abnormal blood vessel occlusion, has shown positive results for PDA closure in premature infants. It is made of a nitinol framework covered partially by a polytetrafluoroethylene membrane at the proximal portion, and it is delivered through a microcatheter with two sizes of 5.3 and 6.5 mm. However, there are two major issues with this device: the unconstrained and constrained lengths (unconstrained length 12 mm for MVP-3Q and MVP-5Q and 16 mm for MVP-7Q) are rather long, and there is a general lack of radio-opacity, making visualization difficult [9]. The Micro Plug is a new microcatheter-delivered device that is currently not commercially available in the European market. Although there are limited published experiences so far, promising safety and efficacy results have been reported in 8 [23] and 25 patients [24], respectively. The APO is currently the only dedicated device for this procedure; it has a particular design for fetal ductus morphology, and an elongated-tubular PDA with a narrowing on the pulmonary side (Hockey stick morphology). It has been CE-marked and FDA-approved for use in ≥700 g and ≥day 3 of life premature infants with a ductal length of ≥3 mm and a minimal ductal diameter of ≤4 mm. It is a self-expandable, Nitinol mesh device with a central cylindrical waist and low-profile retention discs on both ends that are marginally larger than the waist, resulting in a nearly isodiametric device. The device comes pre-loaded on a delivery wire. It can be delivered through a 4-French Amplatzer TorqVue LP catheter (Abbott Structural Heart, Plymouth, MN, USA). The APO is available in nine sizes comprised of three waist diameters (3, 4, and 5 mm) and three lengths (2, 4, and 6 mm).

## 5. Description of the Procedure

**Figure 1:**
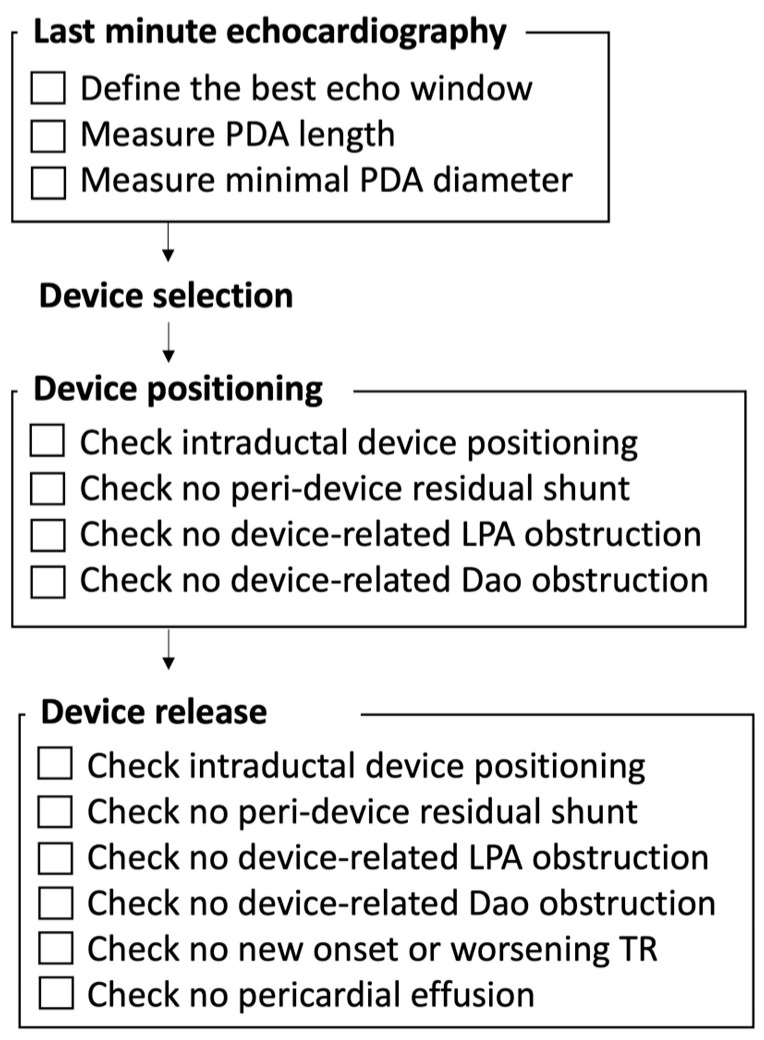
Summary of the main steps of echocardiographic guidance of the procedure.

**Figure 2:**
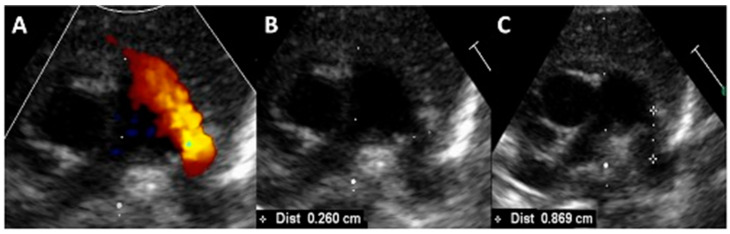
Last minute, pre-procedural echocardiography showing a large, left-to-right shunting, patent ductus arteriosus in an ex-24-week premature infant, with a procedural weight of 740 g (**A**). The minimal ductal diameter was measured at 2.6 mm (**B**), with a ductal length of 8.7 mm (**C**).

Transcatheter PDA closure is usually performed in the cardiac catheterization laboratory under general anesthesia, with the patient connected to his/her own ventilator. High-frequency jet ventilation, when required, does not compromise the device placement success rate [25]. The procedure is performed using both biplane fluoroscopic and transthoracic echocardiographic guidance. The main echocardiographic steps are summarized in Figure 1. A 4-French sheath is inserted in the femoral vein using the standard Seldinger technique, with ultrasound-guided access of the femoral vessel, which reduces the risk of local complications, especially the inadvertent puncture of the femoral artery. Prophylactic antibiotics are administered, but consensus has not been reached on heparin administration; some operators give 50–100 units/kg of unfractionated heparin bolus once access has been achieved [8,10], whereas others only use saline, heparinized, to flush the catheters before insertion with no direct administration of heparin into the patient [8,9]. Patients are enveloped in a warm blanket, with continuous temperature monitoring. In order to shorten the procedure as much as possible, only noninvasive hemodynamic data are collected during the catheterization. A last-minute echocardiographic assessment is made on the table, to note the best echocardiographic window and to measure again the ductal length and the minimal ductal diameter (Figure 2). A catheter led by a soft 0.014 in. wire is advanced from the femoral vein through the right heart and positioned across the PDA into the descending aorta. In the case of an interrupted or obstructed inferior vena cava, a transjugular venous approach for Piccolo PDA closure has been safely reported in VLBW infants [26].

Various techniques are used to position under fluoroscopic guidance the 4-French TorqVue LP delivery catheter over the PDA in the descending aorta. Some interventional teams use a 3.3-French Mongoose catheter (PediaVascular, Chagrin Falls, OH, USA) or a 3.0-French multipurpose BALT catheter (Montmorency, France) over a wire (a 0.018 hydrophilic wire (Terumo Ann Arbor, MI, USA) or a 0.014 soft coronary wire) to advance from the 4 Fr sheath in the femoral vein through the inferior vena cava towards the right atrium and right ventricle. The PDA is crossed, and the catheter is positioned in the descending aorta. Sathanandam et al. have reported a similar technique using a 4-french-angled glide catheter (Terumo, Japan) and a 0.035″ Wholey wire (Medtronic, Minneapolis, MN, USA) to cross the PDA antegradely into the descending aorta [27]. Other teams have used a 4-French Swan-Ganz catheter instead [17]. Sometimes, the 4-French TorqVue LP delivery catheter can be directly advanced over a 2.7-French Progreat microcatheter and a coronary wire in a telescopic fashion [8]. Alternatively, some use a 2.7-French Progreat microcatheter inserted in a 4-French Judkins right coronary catheter [28]. If a 3F catheter is used to cross the PDA, then a 0.021 Fixed-Core Guide Wire (Cook Medical, Bloomington, IN, USA) is advanced in the descending aorta and the 3F catheter is directly exchanged with the 4Fr TorqVue delivery sheath. The tip of the TorqVue sheath is placed in the descending aorta, slightly lower than the PDA.

A hand angiogram of 1.5–2 cc is usually performed in the right anterior oblique and lateral projection, either through the catheter used to cross the PDA or through the TorqVue sheath. In some centers, no contrast is injected and the appropriate device is selected based on procedural echocardiographic measurements of length and minimal ductal diameter only [8,9]. As per the manufacturer’s instructions for use, the device diameter is selected to be 1 mm larger than the PDA diameter, with the device length depending on the PDA length (2 mm length APO if PDA length < 12 mm, 4 mm APO length if PDA length < 12 mm) to avoid protrusion of the device in the descending aorta and/or in the left pulmonary artery [29].

**Figure 3:**
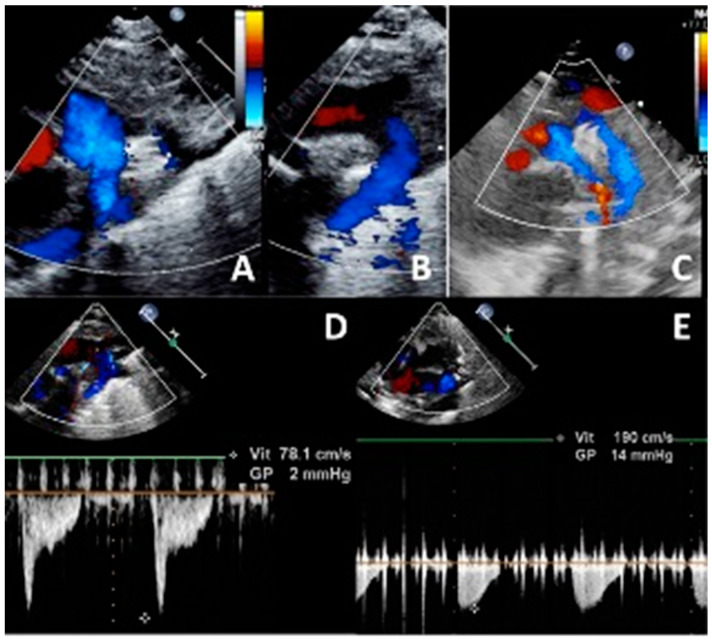
Ultrasound check of a well-positioned 4/2 Amplatzer Piccolo Occluder device, with no residual shunt (**A**) and no device-induced left pulmonary artery obstruction (**A**,**C**,**E**; Vmax 1.9 m/s) or descending aorta obstruction (**B**–**D**; Vmax 0.8 m/s). Note that the procedure is performed on an infant on high-frequency jet ventilation (same patient as Figure 2).

Device deployment and positioning is performed using echocardiography and/or fluoroscopy. As a rule, the device is entirely placed intraductally in infants weighing less than 2000 g, avoiding the uncovered PDA segment at the pulmonary end [30]. Successful positioning is defined by the complete occlusion of the duct with no peri-device residual shunting, and the absence of aortic or LPA obstruction by echocardiography (Figure 3). Following the release of the device, careful echocardiographic assessment is repeated, paying particular attention also to the tricuspid valve function and to the pericardium. The patient is then transferred back to the neonatal intensive care unit for close clinical monitoring and follow-up echocardiography within the first post-procedural 24 h.

## 6. Procedural Efficacy

Transcatheter PDA occlusion in premature infants ≤ 2000 g using the APO device has been reported to be highly efficient, with a >98% success rate in recent large-scale series, including the multicenter American prospective trial (N = 100 patients, procedural weight: 1250 +/− 350 g, success rate: 99%) [10], the multicenter French cohort study (N = 102 patients, procedural weight: 1543 +/− 698 g, success rate: 99%) [9] and a multicenter international study (N = 68 patients, procedural weight: 1200 +/− 370 g, success rate: 98%) [8]. Premature infants did not experience post-PDA ligation syndrome after transcatheter PDA occlusion [31] and had the fastest respiratory improvement and shorter mechanical ventilation when compared to matched cases with surgical PDA ligation [6,8,27]. Moreover, if transcatheter PDA closure was performed before the end of the fourth week of life, the respiratory improvement was even more pronounced [27] and babies were discharged home earlier [8].

## 7. Procedural Safety

In surgical PDA ligation, postoperative complications have been described in up to 44% of ELBW infants, including but not limited to: post-ligation cardiac syndrome (PLCS, >30%), vocal cord paralysis (10–15%) and prolonged mechanical ventilation [32,33,34]. PLCS is defined as the need for hemodynamic support secondary to instability in the first 24 h after surgical or catheterization-based intervention. VLBW infants who undergo transcatheter PDA closure experience significantly lower PLCS rates and have less escalation of respiratory support compared with infants who undergo surgical ligation [31].

Conversely, major procedural complications are infrequent with transcatheter PDA closure in ELBW infants in experienced centers. In the overall experience, procedural-related mortality is very low (Table 1; 1/347 patients, 0.3% mortality rate), and is probably associated with the learning curve in historical series. In 2017, Morville et al. reported one procedural death out of 32 patients, due to a cardiac perforation in a 680 g infant [5]. No procedural deaths but 318 successful APO implantations in <2 kg infants have been reported since then [6,7,8,9,10,11].

Procedure-related MAE should be promptly recognized and appropriately managed. They mainly consist of device embolization (2.3%, percutaneous device retrieval in all cases), device-induced aortic obstruction (1.4%), device-induced LPA obstruction (2.0%) and cardiovascular injury (0.9%). The significant acquired obstruction of adjacent vessels (i.e., descending aorta and/or LPA) is defined by the increased flow velocity >2.5 m/s, as lower flow disturbances have been shown to gradually improve along with vessel growth [35].

**Figure 4:**
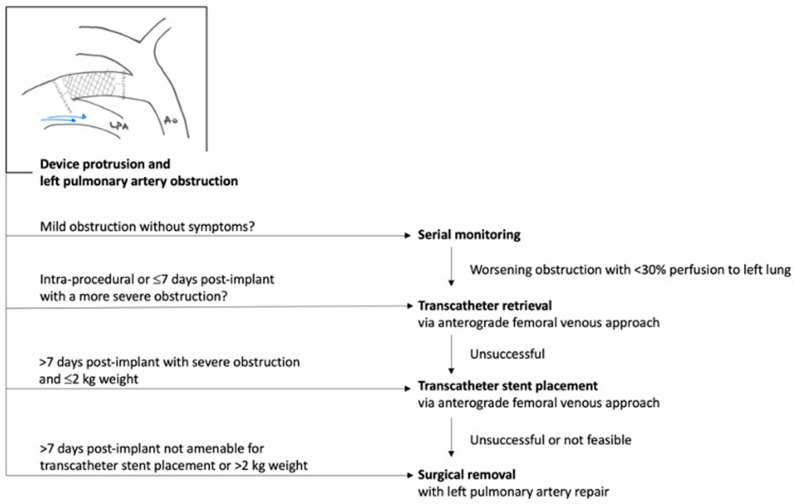
Management algorithm for device-induced left pulmonary artery obstruction. Adapted from consensus guidelines, Sathanandam et al. Pediatr Cardiol. 2021 [4].

**Figure 5:**
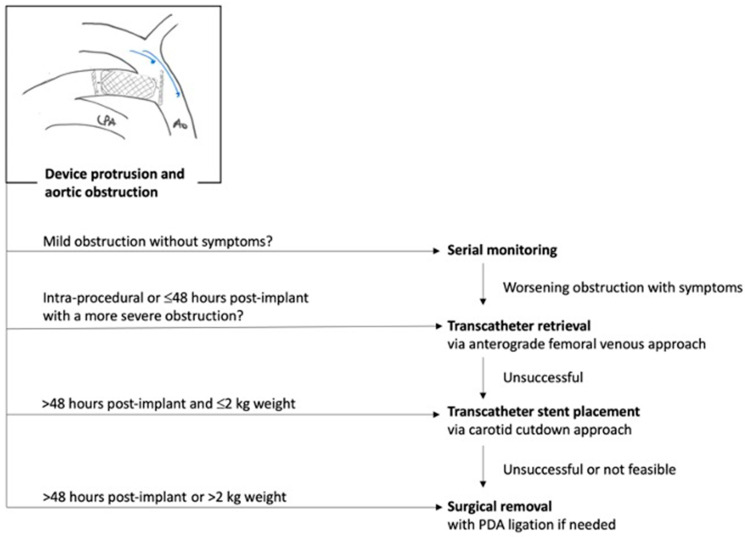
Management algorithm for device-induced aortic obstruction. Adapted from consensus guidelines, Sathanandam et al. Pediatr Cardiol. 2021 [3].

It is noteworthy that along with growing experience, the rate of MAE significantly decreases, dropping from a 10–15% rate of MAE in series <30 patients [5,6,7,11] to a 4–5% rate of MAE in series >60 patients [8,9,10] (Table 1). The incidence of new onset or worsening of tricuspid valve regurgitation in <2 kg infants was 5% in the premarket trial [10] and 4.1% in the French multicenter study [9]. The most common cause of tricuspid regurgitation is injury to the chordae of the septal leaflet of the tricuspid valve, which may become damaged during the catheter crossing of the valve. In the long-term, patients continue to display persistent, unchanged (mild or severe) tricuspid regurgitation without clinical consequences [36]. The incidence, mechanisms and guidelines for the prevention and management of major procedural complications has been summarized in a recent expert consensus statement [4]. Algorithms for the management of device-induced left pulmonary obstruction or aortic obstruction are summarized in Figure 4 and Figure 5, respectively. In the long term, Morray et al. reported high rates of PDA closure and survival of >95% at 3 years with 9/200 reported deaths that were not adjudicated as device- or procedure-related [36].

**Perspectives.** Transcatheter PDA closure is likely to become available to a larger number of preterm ELBW infants. As catheters and delivery systems become increasingly miniaturized, it is anticipated that further improvements, including additional FDA-approved devices and fluoroscopy-free percutaneous approaches, will keep improving the safety and efficacy of transcatheter PDA closure in ELBW infants. Developing a fluoroscopy-free intervention at the patient’s bedspace, without angiography and with echocardiographic guidance only, would be a major step forward in the management of these very preterm infants, in whom the benefit–risk balance would be in favor of avoiding the potential risks associated with both irradiation and contrast injection. Although radiation reduction protocols according to the ALARA concept aim to provide the lowest possible radiation dose, increased radiosensitivity in young children, higher heart rates, smaller cardiovascular structures, and smaller body sizes remain specific challenges, often resulting in relatively high radiation doses to the patient and the possibility of further developing radiation-related sequelae, including increased standardized incidence ratios for all-cancer, leukemia, lymphoma, and solid cancers compared with the general population [37]. The use of a contrast agent in extremely low-birth-weight infants may also cause renal failure or iodine-induced hypothyroidism that has been occasionally reported in ELBW infants after transcatheter PDA closure [38]. In 2011, Bentham et al. reported successful fluoroscopy-free device PDA closure on three premature infants, although the duct was closed from the arterial side [39]. More recently, bedside transcatheter PDA closure solely guided by echocardiography within the neonatal intensive care unit environment has been successfully reported in a 790 g ELBW infant [40] and in a consecutive series of eleven premature infants between 800 and 1600 g [41]. If proved to be efficient and safe in larger patients’ series, this new technique, avoiding both X-ray irradiation and the transportation of these fragile patients to the catheterization laboratory, has the potential to become the basis of a new gold standard for PDA closure in preterm infants.

## 8. Conclusions

In experienced centers, transcatheter PDA closure in ELBW and VLBW infants is technically feasible, with high PDA occlusion success rates and low complication rates in unselected premature infants. Although follow-up studies report favorable short- and medium-term outcomes, they need to be confirmed in prospective trials comparing the results and outcomes of this technique vs. current treatment strategies including medical treatment. This innovative technique is being adopted in an increasing number of centers across the globe; However, further experience is needed. There is an urgent need for multicenter studies and registries to better clarify the results and optimal timing for this procedure, and to study the short-term and long-term outcomes before this can be considered as an alternative to first-line therapy, when PDA closure is required in ELBW infants.

## Figures and Tables

**Table 1:** Procedural safety of transcatheter patent ductus arteriosus in <2000 g premature infants using the Amplatzer Piccolo Occluder.

Author, Year	N	Country	Procedural Weight (kg)	Success Rate (%)	Major AE (%)	Device Embolization	Aortic Obstruction	LPA Obstruction	Cardiovascular Injury	Procedural Mortality
Morville, 2017 [5]	32	France	1.37 (0.68–2.48)	31/32 (97%)	9.4	0 (0.0%)	0 (0.0%)	1 (3.1%)	1 (3.1%) ^a^	1 (3.1%)
Rodriguez, 2018 [6]	27	Spain	1.26 (1.00–1.98)	27/27 (100%)	11.1	2 (7.4%)	0 (0.0%)	0 (0.0%)	1 (3.7%) ^a^	0 (0.0%)
Pamukcu, 2018 [7]	26	Turkey	1.39 (0.75–2.00)	22/26 (85%)	15.4	2 (7.7%)	1 (3.8%)	0 (0.0%)	1 (3.8%)^a^	0 (0.0%)
Regan, 2020 [8]	64	UK, France	1.20 (1.02–1.70)	63/64 (98%)	4.7	2 (3.1%)	1 (1.6%)	0 (0.0%)	0 (0.0%)	0 (0.0%)
Milani, 2020 [9]	73	France	<2000 g	73/73 (100%)	4.1	0 (0.0%)	0 (0.0)	3 (4.1)	0 (0.0%)	0 (0.0%)
Sathanandam, 2020 [10]	100	USA	1.25 (0.7–2.00)	99/100 (99%)	4.0	2 (2.0%)	2 (2.0%)	0 (0.0%)	0 (0.0%)	0 (0.0%)
Wang, 2021 [11]	25	Taiwan	1.21 (0.48–1.98)	25/25 (100%)	16.0	0 (0.0%)	1 (4.0%)	3 (12.0%)	0 (0.0%)	0 (0.0%)
All	350		<2000 g	340/347 (98%)		8/347 (2.3%)	5/347 (1.4%)	7/347 (2.0%)	3/347 (0.9%)	1/347 (0.3%)

Studies were selected if >20 cases had antegrade delivery of an Amplatzer Piccolo Occluder. The Amplatzer Piccolo Occluder is the only device reported in Table 1, because this is currently the only minimally invasive PDA closure device that is FDA approved and CE marked for premature infants. AE: clinically relevant, procedure-related adverse event; USA: United States of America; UK; United Kingdom; ^a^ cardiac tamponade (adapted from Sathanandam et al. Pediatr Cardiol 2021 [4]).
